# Atypical Peripheral Retinal Necrosis Without Ocular Inflammation and Retinal Whitening in a Patient With Acute Myelogenous Leukemia: A Case Report

**DOI:** 10.7759/cureus.54169

**Published:** 2024-02-14

**Authors:** Yoshihiro Nakagawa, Xue Tan, Hikari Yoshida, Takahiro Suzuki, Yasuyuki Suzuki

**Affiliations:** 1 Ophthalmology, Tokai University Hospital, Isehara, JPN

**Keywords:** immunocompromised hosts, retinal necrosis, necrotizing herpetic retinopathy, progressive outer retinal necrosis, acute retinal necrosis

## Abstract

Retinal necrosis is a severe condition that threatens visual function. It is caused by viruses that are known to cause acute retinal necrosis (ARN) and progressive outer retinal necrosis (PORN), which are called necrotizing herpetic retinopathies (NHR). ARN causes severe intraocular inflammation, including anterior chamber intravitreal cells, keratic precipitate, vitreous opacity, and retinal vasculitis, whereas intraocular inflammation in PORN is considered mild or virtually absent. In addition, PORN is a disease that manifests in immunosuppressive patients, such as those with acquired immunodeficiency syndrome.

Here, we present a case of unilateral retinal necrosis after chemotherapy, allogeneic peripheral blood stem cell transplantation, and cord blood transplantation for acute myelogenous leukemia (AML) in a 31-year-old male patient. AML treatment resulted in metabolic remission, and oral steroids and tacrolimus were continued. After two days, the patient visited an ophthalmologist because he noticed a sudden onset of floaters and visual field disturbance in the left eye. The peripheral retina was already necrotic in all layers, causing total retinal detachment. Intraocular inflammation, retinal opacity, or hemorrhagic spots in the fundus were not observed. His previous CD4 count was 43 cells/µL. A polymerase chain reaction test of the anterior chamber fluid revealed varicella-zoster virus (VZV), and vitrectomy was performed four days after disease onset. The excised vitreous demonstrated minimal opacity. The peripheral necrotic retina was excised, photocoagulation was performed on the residual retinal limbus, and silicone oil was injected to maintain retinal attachment. The retinal restoration was maintained under silicone oil tamponade, and corrected visual acuity improved to 20/32 without strong inflammation after vitrectomy. However, two months postoperatively, he contracted coronavirus disease 2019 (COVID-19), his general condition rapidly deteriorated, and he died.

This case of retinal necrosis without inflammatory results in an immunocompromised patient and VZV detection in an intraocular sample led us to suspect PORN. However, the patchy or spread retinal whitening characteristic of PORN was completely absent, whereas the well-defined, peripheral, full-layer retinal necrosis characteristic of ARN was present. Thus, this is a rare case of VZV-induced NHR with partial features of PORN and ARN that progressed very silently.

## Introduction

Bacterial endophthalmitis or viruses cause retinal necrosis. Most cases caused by viruses are due to herpesviruses such as the herpes simplex virus (HSV), varicella-zoster virus (VZV), cytomegalovirus (CMV), and Epstein-Barr virus (EBV) [1,2]. Herpetic retinal necrosis (NHR) is relatively rare, but retinal function, which is a part of the sensory organs, is frequently impaired by necrosis, and retinal detachment often occurs, leaving significant visual impairment [3]. Acute retinal necrosis (ARN) is more prominent in immunocompetent individuals, whereas progressive outer retinal necrosis (PORN) is more prevalent in patients with immunosuppression, such as those with acquired immunodeficiency syndrome (AIDS) [4]. However, many studies have reported that the immune status is not as described [5,6], and a concept recommended that ARN and PORN are diseases of the same spectrum [7].

Here, we report a rare case of NHR with peripheral retinal necrosis from the onset but with no intraocular inflammatory findings, hemorrhagic spots, or white opacity in the retina.

## Case presentation

A 31-year-old male patient with acute myelogenous leukemia (AML) diagnosed five years ago relapsed after inducing remission and consolidation therapy with cytarabine. Therefore, the patient underwent human leukocyte antigen-haploidentical peripheral blood stem cell transplantation four years ago. Subsequently, the patient had another relapse and underwent cord blood transplantation, which resulted in a complete response (CR). However, three years ago, he developed post-transplant lymphoproliferative disorder, and metabolic CR after adding CHASER (cyclophosphamide, high dose Ara-C, steroid, etoposide, and rituximab) therapy. At the time of cataract surgery, the patient was taking 10 mg of methylprednisolone and 1 mg of tacrolimus per day for about six months. One month before the onset of retinal necrosis, methylprednisolone was increased to 16 mg per day, and tacrolimus was decreased to 0.5 mg per day.

**Figure 1:**
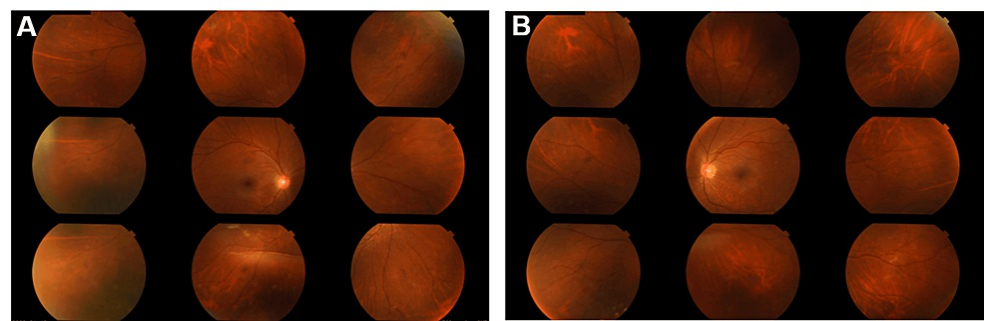
Seven months before the onset of retinal necrosis (A and B) Nine-directional fundus photographs. Both eyes demonstrated no retinal abnormalities.

His previous ophthalmologic history indicated cataract surgery one year earlier due to bilateral posterior subcapsular cataracts, probably caused by steroids, and an intraocular lens had been inserted into the capsule. The refraction, which was originally -5 D, was reduced to -3 D. The postoperative course was good, the best corrected visual acuity was 20/20 in both eyes three months postoperatively, and the fundus was clear with no abnormal retinal findings (Figure 1).

**Figure 2:**
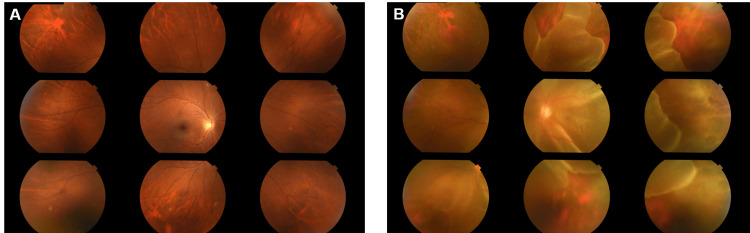
Nine-directional fundus photographs at the onset of retinal necrosis (A) The fundus of the right eye is clear and exhibits a retinal tear on the temporal superior retina. (B) Left eye. The peripheral retina demonstrated well-defined thinning and total detachment of the retina, including the posterior pole.

At this time (one year after cataract surgery), he visited our hospital with complaints of floaters in his left eye two days earlier and loss of visual field one day earlier. The best corrected visual acuity was 20/20 in the right eye and 20/100 in the left eye. As for his general condition, there were no particular major changes, but he received occasional blood transfusions due to chronic anemia, and his hemoglobin level fluctuated between 6.1 and 10.1 g/dL for the past six months. Also, his previous CD4-positive cell count was 43 cells/mL. He had no complaints of ocular pain and no conjunctival hyperemia or inflammatory changes in the anterior segment of both eyes under slit-lamp microscopy. Fundus examination under mydriasis revealed a retinal tear in the right retina on the temporal superior side but no retinal detachment. The left eye demonstrated total retinal detachment with no hemorrhagic or exudative spots. The peripheral retina was atrophic in a mesh-like pattern and bordered clearly with the retina that had not atrophied (Figure 2).

A vitrectomy was performed four days after the onset. The fundus was clearly visible without vitreous opacity under microscopy (Video 1). The posterior vitreous detachment had already occurred, and there were no tractional changes in the vitreous and retina. The atrophic retina was resected, photocoagulation was performed on the limbus of the remaining retina, and silicone oil was injected to keep retinal attachment.

**Video 1:** Intraoperative retinal findings Total retinal detachment was observed in the left eye, and thinning of the peripheral retina due to necrosis was directly visible. The peripheral necrotic retina was circumferentially trimmed using triamcinolone acetonide and perfluorocarbon liquid.

**Figure 3:**
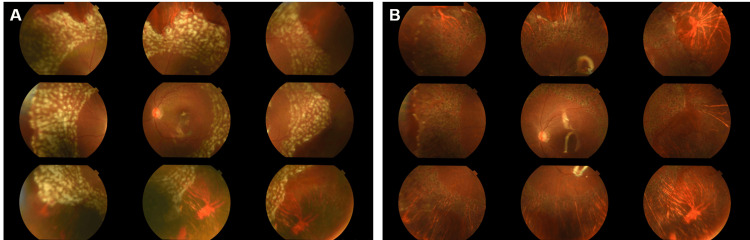
Nine-directional fundus photographs of the left eye after vitrectomy (A) One day postoperatively. The retina can maintain its reposition under silicone oil tamponade. (B) One month postoperatively. Retinal attachment was maintained, and the photocoagulated spots were scarred.

The comprehensive polymerase chain reaction (PCR) test of the anterior chamber fluid collected intraoperatively was positive for VZV and negative for HSV, CMV, and EBV. The bacterial culture of the same fluid was negative. Postoperative medication was discussed with the hematologist because there were no intraocular inflammatory findings, and steroids and tacrolimus were continued. Postoperatively, the retinal attachment was maintained under silicone oil tamponade, and best corrected visual acuity recovered to 20/32 (Figure 3A). Retinal photocoagulation was performed under a slit-lamp microscope for the retinal tear in the right eye with no other abnormal results. One month postoperatively, no intraocular inflammation was observed in either eye, with no new retinal detachment (Figure 3B).

Two months following vitrectomy, the patient developed a fever of 39℃, which was the first symptom of coronavirus disease 2019 (COVID-19). He was hospitalized and treated; however, his general condition deteriorated with the appearance of hemorrhagic stroke and decreased level of consciousness, and he died approximately three months after vitrectomy.

## Discussion

This patient demonstrated a low count of CD4-positive cells and was in an immunosuppressive state because of AML and its treatment. The PCR test of the anterior chamber fluid detected VZV, but other members of the *Herpesviridae* family tested negative. In addition, bacterial culture of the anterior chamber fluid exhibited negative results, causing us to consider this case as VZV-associated retinal necrosis. As noted in the third paragraph of the Case Presentation, the changes in peripheral retinal necrosis, in this case, were remarkably prominent; however, signs of intraocular inflammation were not observed, and subjective symptoms appeared only upon the occurrence of retinal detachment. This makes it a rare case where we could not find a reported similar course in NHR.

VZV-associated retinal diseases include ARN and PORN. ARN, also known as Kirisawa’s uveitis, was first reported by Urayama in 1971 [8], while Forster et al. were the first to report two cases of outer retinal necrosis in immunocompromised patients, defining PORN [9]. Austin described them as being at opposite ends of the spectrum of NHR, detailing their respective characteristics [10]. Regarding subjective symptoms, PORN typically manifests with sudden vision loss and visual field defects, whereas ARN presents with hazy vision. In terms of intraocular inflammation, patients with PORN exhibit little to no inflammation, whereas patients with ARN frequently demonstrate significant inflammation in the anterior segment and vitreous. Retinal findings include a “cracked mud appearance” characteristic of PORN in the peripheral or posterior pole, whereas ARN demonstrates retinal vasculitis outside the vascular arcade. In this case, on the basis of subjective symptoms, inflammatory findings, and immune status, PORN was the most likely suspicion. However, Engstrom et al. observed white retinal lesions in all 65 cases of PORN, which were not observed in this case [11]. The most intense change revealed was peripheral retinal necrosis characterized by a well-defined necrosis of the entire peripheral retinal layer, which aligns more closely with ARN. ARN typically presents unilaterally, with a few cases of bilateral involvement reported, whereas PORN often progresses to affect both eyes despite initial onset in one [11,12]. In our case, only the left eye exhibited NHR, while the retinal tear in the right eye was attributed to myopia. Although the diagnosis seems closer to ARN due to the absence of binocular involvement, the patient passed away two months after onset, leaving the long-term monocular nature of the disease unknown.

NHR is closely associated with the patient’s immune status [4]. ARN cases are found in immunocompromised individuals, whereas it frequently occurs in immunocompetent patients. Conversely, PORN is more prevalent in immunosuppressed patients, such as those with AIDS. Lewis et al. and Klecicka et al. reported PORN cases after renal transplantation and after bone marrow transplantation, respectively [13,14]. However, Chawla et al. and Carrillo-Pacheco et al. reported PORN cases in immunocompetent patients, emphasizing the difficulty of clearly distinguishing between ARN and PORN [6,15].

Regarding the postoperative course, we achieved visual acuity recovery without intravitreal and intravenous injections of antiherpetic drugs, and retinal restoration was maintained. Fitoussi et al. reported a 1.3 (logMAR) mean corrected visual acuity three months after onset in 37 patients with NHR. In comparison, our patient demonstrated good visual acuity, likely due to the silent disease course with no inflammation, retinal hemorrhage, or white opacity other than retinal necrosis [1].

## Conclusions

Our patient developed VZV-induced unilateral NHR and achieved excellent visual recovery with vitrectomy. No intraocular inflammatory signs were observed, and subjective symptoms did not manifest until retinal detachment. Our case has some of the ARN and PORN features but does not fit the diagnosis of either and may demonstrate a disease state exactly in the middle of the spectrum, where peripheral retinal necrosis progressed remarkably silently.
